# A report on the potential of Rac1/pSTAT3 protein levels in T lymphocytes to assess the pharmacodynamic effect of thiopurine therapy in Inflammatory Bowel Disease patients

**DOI:** 10.1038/s41598-022-20197-5

**Published:** 2022-09-22

**Authors:** Debbie S. Deben, Rob H. Creemers, Arjan J. van Adrichem, Roosmarie Drent, Audrey H. H. Merry, Mathie P. G. Leers, Adriaan A. van Bodegraven, Dennis R. Wong

**Affiliations:** 1 Department of Clinical Pharmacy, Clinical Pharmacology and Toxicology, Zuyderland Medical Centre, Dr. H. van der Hoffplein 1, 6162 BG Sittard-Geleen, The Netherlands; 2 Department of Gastroenterology, Geriatrics, Internal and Intensive Care Medicine (Co-MIK), Zuyderland Medical Centre, Sittard-Geleen/Heerlen, The Netherlands; 3 Department of Clinical Chemistry and Haematology, Zuyderland Medical Centre, Sittard-Geleen/Heerlen, The Netherlands; 4 Department of Internal Medicine, Division of Gastroenterology and Hepatology, Maastricht University Medical Centre+, Maastricht, The Netherlands; 5 Accureon BV, Clinical Chemistry and Hematology Laboratory, Roosendaal, The Netherlands; 6 Zuyderland Medical Centre, Zuyderland Academy, Sittard-Geleen/Heerlen, The Netherlands; 7 Department of Gastroenterology and Hepatology, Amsterdam University Medical Centre, Amsterdam, The Netherlands

**Keywords:** Inflammatory bowel disease, Diagnostic markers

## Abstract

The thiopurine derivatives azathioprine (AZA), mercaptopurine (MP) and tioguanine (TG) remain standard treatment of inflammatory bowel disease (IBD). The immune suppressive effect of thiopurines is primarily based on blocking the Ras-related C3 botulinum toxin substrate 1 (Rac1) causing apoptosis of T lymphocytes by inhibition of the phosphorylated downstream transcription factor Signal Transducer and Activator of Transcription 3 (pSTAT3). A functional pharmacodynamic marker in T lymphocytes may be useful to predict therapeutic outcome of thiopurine therapy. The aim of this study was to explore whether protein levels of Rac1 and pSTAT3 in T lymphocytes may be applied as a specific pharmacodynamic marker for thiopurine therapy in IBD patients. Rac1 and pSTAT3 protein levels in T lymphocytes were explored in 57 IBD patients (median age 51 years, 56% female), subdivided into six groups based on IBD activity and its treatment: patients with active disease without IBD maintenance medication (1) or patients in remission on AZA/MP (2), TG (3), infliximab (IFX) (4), thiopurine and IFX combination-treatment (5) or without IBD medication (6). Reference values were obtained from healthy subjects. Rac1 and pSTAT3 protein levels in T lymphocytes from patients on thiopurine monotherapy (group 2 and 3) were compared to the other groups, and to healthy subjects. Absolute Rac1 and pSTAT3 protein levels showed no differences between the thiopurine monotherapy groups when compared to patients with active disease. However, the ratio of Rac1 and pSTAT3 protein levels was lower in thiopurine patients groups compared to patients with active disease. Rac1-corrected pSTAT3 protein levels may serve as a pharmacodynamic marker of thiopurine monotherapy and may be a potential tool to predict therapeutic effectiveness in IBD patients.

## Introduction

Crohn’s disease (CD) and ulcerative colitis (UC), the two main phenotypes of inflammatory bowel disease (IBD), are chronic and relapsing inflammatory diseases of the digestive tract. The incidence and prevalence of IBD are rising worldwide^1,2^.

Thiopurine derivatives have successfully been used in IBD treatment over 50 years with proven effectiveness in (inducing and) maintaining remission in up to 75% in CD and UC patients^3–5^. Over the past decades, new biological treatment options for IBD have also become widely available. However, to date, thiopurines are still first choice of immune suppressive maintenance treatment in patients with moderate-to-severe IBD^5^. Worldwide, thiopurines are increasingly being prescribed, especially in the majority of countries without or with limited access to costly, parenteral, biological therapy^6,7^. Currently applied thiopurine derivatives are the conventional thiopurines azathioprine (AZA) and mercaptopurine (MP), and the alternative thiopurine tioguanine (TG), which is in particular used in IBD patients who previously failed AZA/MP therapy^8,9^.

**Figure 1:**
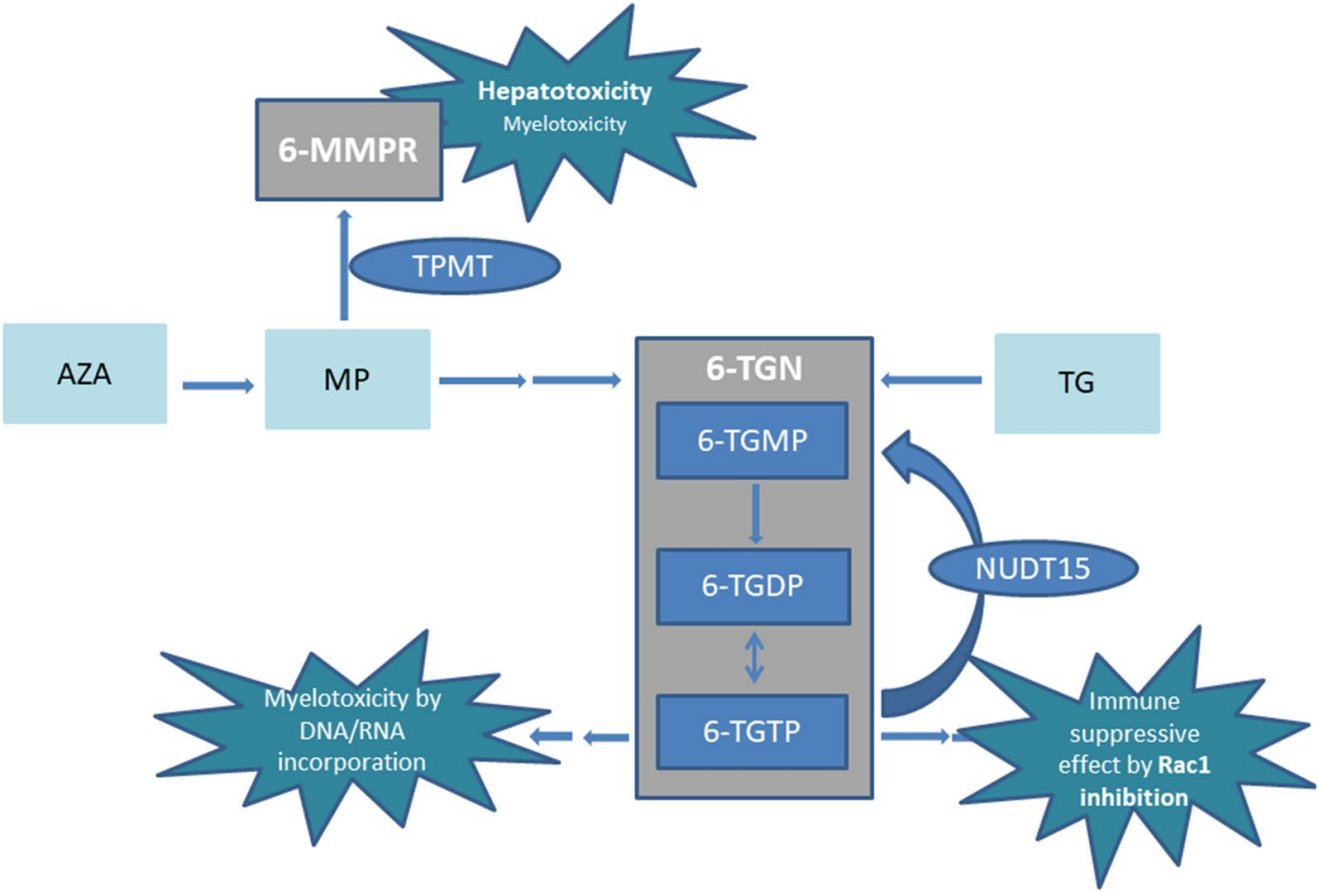
A simplified scheme of thiopurine metabolism. *6-MMPR* 6-methylmercaptopurine ribonucleotides, *6-TGMP* 6-thioguanine monophosphate, 6TGDP: 6-thioguanine diphosphate, *6TGTP* 6-thioguanine triphosphate, *6-TGN* 6-thioguanine nucleotides, *AZA* azathioprine, *MP* mercaptopurine, *NUDT15* Nudix hydrolase 15, *Rac1* Ras-related C3 botulinum toxin substrate-1, *TG* tioguanine, *TPMT* Thiopurine S-Methyl Transferase.

Thiopurine derivatives are extensively metabolised and eventually converted into the 6-thioguanine nucleotides (6-TGN), comprising 6-thioguanine monophosphate (6-TGMP), 6-thioguanine diphosphate (6-TGDP) and 6-thioguanine triphosphate (6-TGTP), of which the latter is believed to primarily contribute to the pharmacological immune suppressive properties (Fig. 1)^10^. Other metabolites of the conventional thiopurine metabolic pathway, specifically the 6-methylmercaptopurine ribonucleotides (6-MMPR), are associated with hepatotoxicity^11,12^.

Unfortunately, thiopurine therapy is known to have a delayed onset of therapeutic response, as clinical response gradually develops within one to six months. Besides the delayed onset of therapeutic response, up to half of patients discontinue conventional thiopurine therapy within two years due to therapeutic failure or intolerable adverse drug events, such as gastrointestinal complaints, malaise and hepatotoxicity^3^.

Optimization of individual therapy is therefore advocated, which is nowadays primarily based on pharmacogenetic and pharmacokinetic strategies. Therapeutic drug monitoring of 6-TGN and 6-MMPR metabolites is widely recommended as a useful pharmacokinetic optimization strategy to explain or prevent adverse drug events and therapeutic non-response due to non-compliance^13–15^.

Furthermore, pharmacogenetic optimization strategies by prior-to-treatment genotyping of the genes encoding thiopurine S-methyltransferase (*TPMT)* and nudix hydrolase 15 (*NUDT15)* have become available to prevent thiopurine-induced leukopenia^16^.

However, by using these pharmacokinetic and -genetic optimization strategies, clinical effectiveness and drug safety can only be partly predicted or improved^17^. It has been suggested that a functional pharmacodynamic marker may be more helpful to monitor or to predict therapeutic outcome of thiopurines^18^.

**Figure 2:**
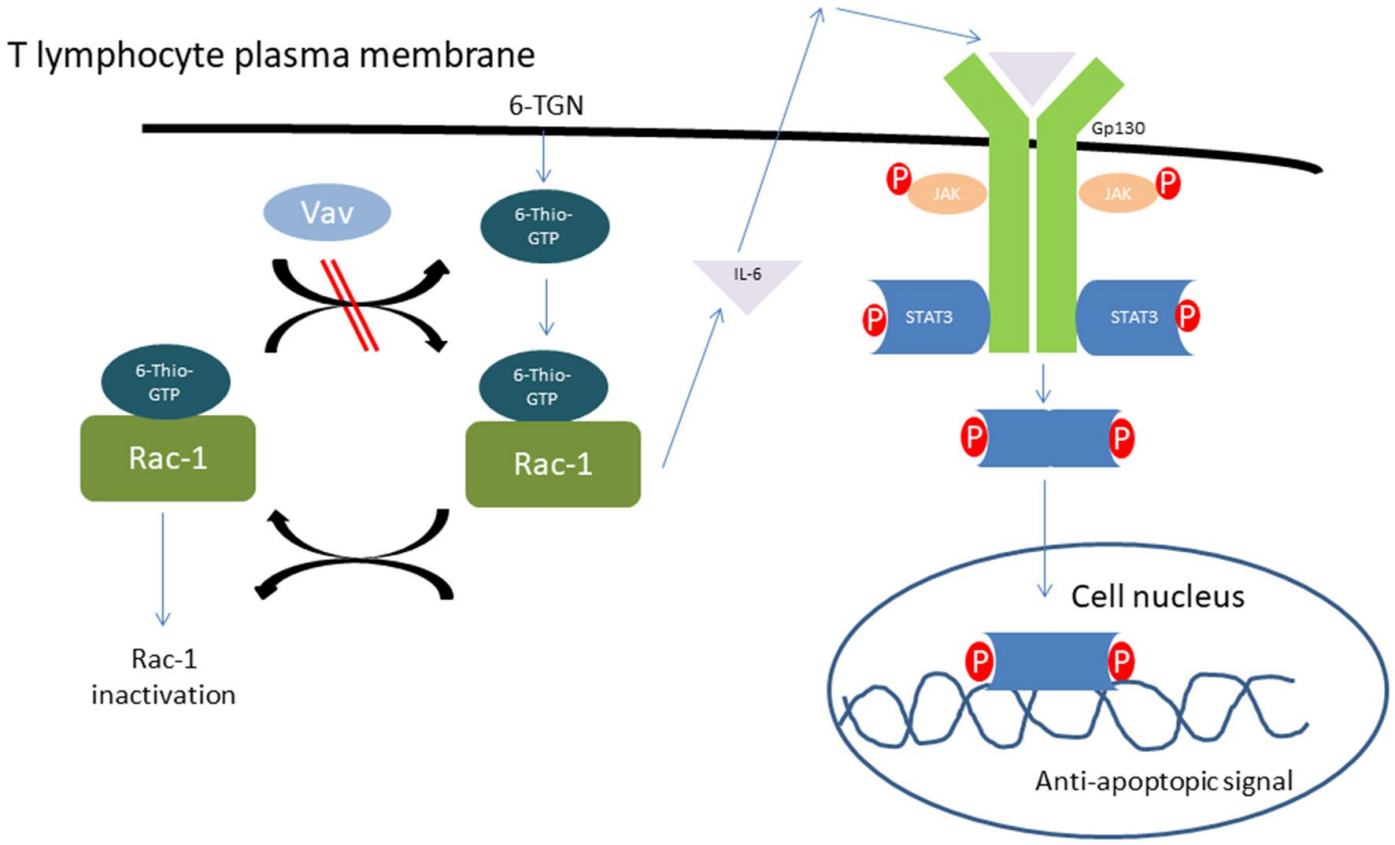
A simplified scheme of the Rac1/pSTAT3 pathway. *6-TGN* 6-thioguanine nucleotides, *6-TGTP* 6-thioguanine triphosphate, *gp130* G-protein 130, *IL-6* Interleukine-6, *JAK* Janus Kinase, *Rac-1* Ras-related C3 botulinum toxin substrate-1, *STAT3* Signal transducer and activator of transcription 3, *Vav* guanine nucleotide exchange factor.

The immune suppressive pharmacodynamic effect of thiopurines in IBD dosages (2–2.5 mg/kg bodyweight for AZA, 1–1.5 mg/kg bodyweight for MP and 0.2–0.3 mg/kg bodyweight for TG) is primarily based on inhibition of the small GTPase Ras-related C3 botulinum toxin substrate 1 (Rac1) causing apoptosis of activated T lymphocytes (Fig. 2)^18^.

Rac1 functions as a rapid molecular switch in the cell, continuously cycling between an inactive (GDP-bound) and an active (GTP-bound) state. In the active state, Rac1 binds to different effector molecules, regulating cell proliferation, cell-cycle progression and activation of various protein kinases, amongst other effects^19^. When the thiopurine metabolite 6-TGTP, instead of physiological GTP, binds Rac1, inherent GTPase activity will cause Rac1-bound-TGTP to be inactivated into Rac1-bound-TGDP. However, the GTP/GDP exchange factor Vav is not able to bind Rac1-bound-TGDP, so that reactivation of Rac1 is blocked and T lymphocyte apoptosis is induced (Fig. 2)^20^.

The role of Rac1 suppression and its downstream effectors in IBD has been studied before^20–22^. Moreover, measuring Rac1 protein levels as a pharmacodynamic marker for thiopurine drugs has previously been described^18,23^. However, exclusively measuring Rac1 fails to provide crucial information on the ‘on/off’-status of the GTPase. Therefore, a combination of measurement of Rac1 and its downstream effector protein Signal Transducer and Activator of Transcription 3 (STAT3) is likely to be clinically more valuable, potentially identifying the pharmacodynamic effect of a successful thiopurine-induced anti-inflammatory effect.

Recently, we developed and validated a functional pharmacodynamic multiparameter flow cytometric assay to determine the protein levels of both Rac1 and phosphorylated (*i.e.* activated) STAT3 (pSTAT3) in various leukocyte subpopulations in peripheral blood of healthy subjects and IBD patients^24^.

The aim of this observational explorative study is to evaluate Rac1 and pSTAT3 protein levels in T lymphocytes of IBD patients with active disease and IBD patients in remission on thiopurine monotherapy. Furthermore, Rac1 and pSTAT3 protein levels were analysed in healthy subjects and in different IBD patient groups, subdivided by the use of various drug regimens.

## Methods

### Study design and patient population

This was a prospective observational single centre explorative study in Zuyderland Medical Centre, one of the largest referral hospitals for IBD care in the Netherlands. IBD patients between 18 and 70 years old were considered eligible for this study. Per drug-classified group (outlined below), inclusion of ten patients was intended.

Exclusion criteria were patients with other auto-immune diseases, use of systemic immune suppressive drugs other than thiopurines and anti-TNF therapy for patients in remission, or proven non-adherence.

The diagnosis of IBD, comprising Crohn's disease, ulcerative colitis and unclassified IBD (IBD-U), was based on the standard combination of clinical, biochemical, endoscopic, radiologic and histological findings. Physician global assessment (PGA) was used to define the clinical disease activity status of UC, CD and IBD-U. Patients were categorized as remission or active disease (mild, moderate or severe). All three categories of active disease were considered eligible for inclusion. In addition, PGA was objectified by biochemistry, and clinical activity scores, comprising Partial Mayo (pMayo) and Harvey-Bradshaw Index (HBI), for UC / IBD-U and CD, respectively^25,26^.

IBD patients were categorized into the following groups: IBD patients with active disease without IBD maintenance medication (except local or systemic corticosteroids) (1) and several drug-classified groups of IBD patients in remission on: AZA/MP monotherapy (2), TG monotherapy (3), infliximab (IFX) monotherapy (4), AZA/MP or TG and IFX combination therapy (5), and patients in remission without immune suppressive medication (6). All patients in remission with medication were on that drug regimen for at least eight weeks. Patients with active disease were included prior to initiation of thiopurine therapy.

Data from healthy subjects were obtained from the previous laboratory validation study and were used as reference values^24^. Healthy subjects were defined by having no known history of auto-immune diseases, no fever or other clinical complaints of active disease, no use of immune suppressive medication and a C-Reactive Protein (CRP) concentration below 10 mg/L.

### Data collection

Medical files of consecutive IBD patients attending the Outpatient Clinic (or the Outpatient Infusion Centre) were screened at least two weeks prior to their visit. Patients who were eligible to participate in the study were invited and blood samples for measurement of Rac1, pSTAT3 and CRP were obtained. Data on demographics, disease activity, medical records and laboratory parameters, such as baseline faecal calprotectin, cell compartments of subpopulations of leukocytes and baseline 6-TGN and 6-MMPR concentrations were collected and registered using data management (Research Manager, version 5.58.0.3). Furthermore, we objectified non-adherence of all patients based on undetectable 6-TGN and/or 6-MMPR baseline concentrations.

### Rac1/pSTAT3 measurement

Peripheral ethylenediamine tetraacetic acid (EDTA) blood samples were used for immunocytochemical labelling, as recently described^24^. In brief, after blood collection, blood cells were washed and prepared for analysis with flowcytometry. All analyses were performed within one hour after venepuncture, since stability of Rac1 protein levels this period has been demonstrated before^24^. First, the cells in suspension were incubated with fluorochrome-labelled antibodies targeting extracellular antigens, followed by addition of Lyse and Fix buffer for erythrocyte lysing and fixation of the antibodies to the membrane-bound antigens. Subsequently, fluorochrome-labelled antibodies targeting intracellular antigens were added and incubated, followed by washing and resuspending cells in 1% bovine serum albumin / phosphate buffered saline (BSA/PBS) buffer for flowcytometric analysis. The samples were analysed using a BD FacsCanto flow cytometer (BD Biosciences, San Jose, USA). Lastly, a gating procedure was performed to differentiate between leukocyte subpopulations (Supplemental Data).

### Data analysis

Rac1 and pSTAT3 protein levels were reported as fraction of positive cells as well as mean fluorescence intensity (MFI). For all IBD patients, total fractions of Rac1 and pSTAT3 protein levels were displayed as normalized MFI based on healthy subjects, reported as *arbitrary units* (AU). Normalized MFI was calculated by dividing an individual’s MFI by the mean MFI of healthy subjects. Conversion of raw data into AU is explained in more detail in the Supplemental Data.

Besides Rac1 and pSTAT3 protein levels, the ratio of pSTAT3 and Rac1 protein levels was calculated. This ratio was used to analyze the proportion of pSTAT3 in relation to the available Rac1. This was introduced because it was hypothesized that the inhibition of Rac1 by thiopurine therapy could in fact induce total (active and inactive) Rac1 protein expression as means of a compensation mechanism. Any potential increase of Rac1 protein expression will be corrected by the use of a ratio of pSTAT3/Rac1. Therefore, relative pSTAT3 protein levels were reported as absolute pSTAT3 protein levels divided by absolute Rac1 protein levels, correcting for absolute (inactive) Rac1 quantities.

Baseline characteristics were presented as medians with interquartile range (IQR) for non-normal distributed numerical variables and as number of patients with corresponding percentage for categorical variables. Independent Kruskal–Wallis test was used for comparison of numerical variables and Chi-square test for categorical variables. Statistical analyses were conducted using SPSS (Version 26.0). P-values of ≤ 0.05 were considered to be statistically significant.

To investigate possible differences in Rac1 and pSTAT3 protein levels between groups, statistical analyses were conducted using GraphPad Prism (version 8.0, San Diego, CA, USA). Unpaired t-tests and Mann–Whitney U tests were used for comparison of normal distributed and non-normal distributed variables, respectively. Because of the Bonferroni-correction for five independent comparisons, a corrected p-value of ≤ 0.01 (p ≤ 0.05 divided by 5) was considered statistically significant for these statistical tests.

### Ethical considerations

The study was approved by the medical ethics committee of Zuyderland medical centre and Zuyd Hogeschool (METC Z, Heerlen, The Netherlands, METC-number METCZ20200110) and conforms to the ethical guidelines of the Declaration of Helsinki (2013). All participants provided written informed consent. Written informed consent was also obtained from healthy subjects.

## Results

### Patient characteristics

**Figure 3:**
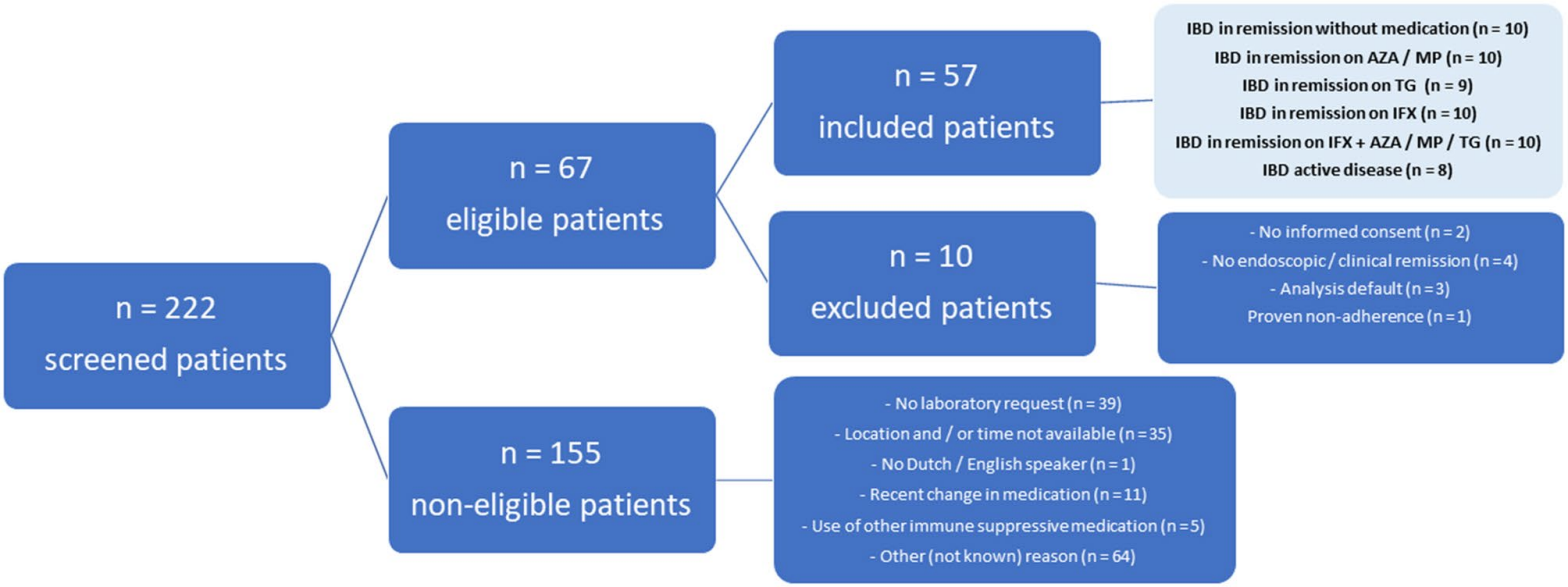
Flowchart of patient inclusion. *AZA* azathioprine, *IBD* inflammatory bowel diseases, *IFX* infliximab, *MP* mercaptopurine, *TG* tioguanine.

In total, 222 IBD patients were identified of which 155 cases were considered non-eligible (Fig. 3). Of all 67 eligible patients, ten patients were excluded because of absence of written informed consent (n = 2), an error in the analysis (n = 3), non-adherence of thiopurine therapy (n = 1) or no clinical/endoscopic remission (n = 4). The 57 remaining patients were subdivided into six different groups, as depicted in Fig. 3.

Baseline characteristics of all groups are presented in Table 1. A total of 32 patients (56%) were female and 33 patients (58%) had CD. Both gender and IBD disease type did not differ between groups (p = 0.907 and p = 0.331, respectively). The median age was 51 years (IQR 33–62) and the median BMI was 24.6 kg/m^2^. Both age (p = 0.331) and BMI (p = 0.618) did not differ between groups.

**Table 1:** Patient characteristics.

	All IBD patients (n = 57)	Active disease (n = 8)	In remission with					*P*-value
			AZA/MP (n = 9)	TG (n = 10)	IFX (n = 10)	IFX + AZA/MP/TG (n = 10)	No therapy (n = 10)	
**Gender**								0.907 +
Females, n(%)	32 (56)	4 (50)	4 (44)	5 (50)	4 (40)	6 (60)	7 (70)	
Males, n(%)	25 (43)	4 (50)	5 (56)	5 (50)	6 (60)	4 (40)	3 (30)	
Age (years), median (IQR)	51 (33–62)	60 (51–65)	46 (33—60)	50 (47–55)	37 (30–66)	36 (24–58)	58 (57–62)	0.331*
BMI (kg/m^2^), median (IQR)^a^	24.6 (23.2–28.7)	24.1 (22.9–26.2) (n = 7)	28.7 (23.3–34.0)	26.4 (23.6–30.1)	24.2 (23.1–30.1) (n = 9)	24.2 (20.4–26.2)	24.3 (23.3–26.9)	0.618*
**IBD disease type**								0.056 +
Ulcerative colitis, n(%)	23 (40)	3 (38)	5 (56)	3 (30)	1 (10)	3 (30)	8 (80)	
Crohn’s disease, n (%)	33 (58)	4 (50)	4 (44)	7 (70)	9 (90)	7 (70)	2 (20)	
IBDU, n (%)	1 (2)	1 (13)						
**Dose**								
AZA (mg/kg), median (range)			0.67 (0.22–1.71)					
MP (mg/kg), median (range)			0.67 (0.57–0.93)			1.48 (1.07–1.89)		
TG (mg/kg), median (range)				0.14 (0.11–0.47)		0.68 (0.63–0.69)		
IFX (mg/kg), median (range)					5.1 (4.0–6.5)	5.1 (4.1–10.9)		
Interval IFX (weeks), median (range)					7 (4–8)	7 (4–8)		
**IBD disease severity**								
pMayo, median (range)		4 (3–8)	0 (0–1)	0 (0–3)	0 (N/A)	0 (0–1)	0 (0–1)	0.008*
HBI, median (range)		6 (5–6)	1 (0–6)	1 (0—2)	0 (0–4)	1 ( 0–4)	0 ( 0–0)	0.029*
PGA								
Remission			9	10	10	10	10	
Mildly active		5						
Moderately active		3						
CRP (mg/L), median (IQR)		4 (2–8)	2 (1–3)	3 (1–6)	2 (1–3)	1 (1–3)	1 (1–2)	0.250*
FCP (mg/kg), median (IQR)^b^		1387 (541–3405) (n = 7)	69 (28–117) (n = 8)	65 (20–118) (n = 8)	55 (14–122) (n = 6)	34 (12–70) (n = 9)	45 (13–60) (n = 9)	0.001*
**Cell compartments**								
T lymphocytes (%), median (IQR)	80.1 (73.4–86.2)	79.5 (70.4–83.2)	89.1 (75.0–93.5)	80.5 (74.1–84.7)	72.9 (66.3–85.6)	89.2 (80.8–90.9)	76.4 (62.9–81.4)	0.127*
B lymphocytes (%), median (IQR)	7.8 (5.1–11.2)	8.0 (5.7–11.0)	3.0 (2.0–9.8)	9.1 (5.7–12.4)	10.6 (5.1–14.7)	7.2 (4.7–11.3)	7.9 (6.8–10.3)	0.469*
NK-cells (%), median (IQR)	10.2 (5.4–16.0)	11.4 (9.4–18.6)	7.9 (3.9–14.9)	9.6 (3.3–17.1)	12.0 (8.4–21.6)	5.0 (3.3–7.5)	14.3 (10.0–26.0)	0.050*

*BMI* body mass index, *IBD* inflammatory bowel disease, *IBD-U* IBD-unclassified, *AZA* azathioprine, *MP* mercaptopurine, *TG* tioguanine, *IFX* infliximab, *HBI* Harvey Bradshaw Index, *NK cells* natural killer cells, *pMayo* partial Mayo score, *PGA* physician’s global assessment, *CRP* C-reactive protein, *FCP* faecal calprotectin, *IQR* interquartile range.

+ Chi-square.

*Kruskal–Wallis.

^a^No BMI available in 2 IBD patients: 1 IBD patient with IFX monotherapy and 1 IBD patient with active disease.

^b^No FCP concentration available in 10 IBD patients: 1 IBD patient with active disease, 1 IBD patient on AZA/MP monotherapy, 2 IBD patients on TG monotherapy, 4 IBD patients on IFX monotherapy, 1 IBD patient on IFX and AZA/MP/TG combination therapy, and 1 IBD patient without immune suppressive medication.

IBD patients in the active disease group differed from all IBD patient groups in remission in both disease activity scores (pMayo score p = 0.008 and HBI score p = 0.029), as well as faecal calprotectin concentration (p = 0.001). CRP did not differ between all groups (p = 0.250).

### Rac1 expression

**Figure 4:**
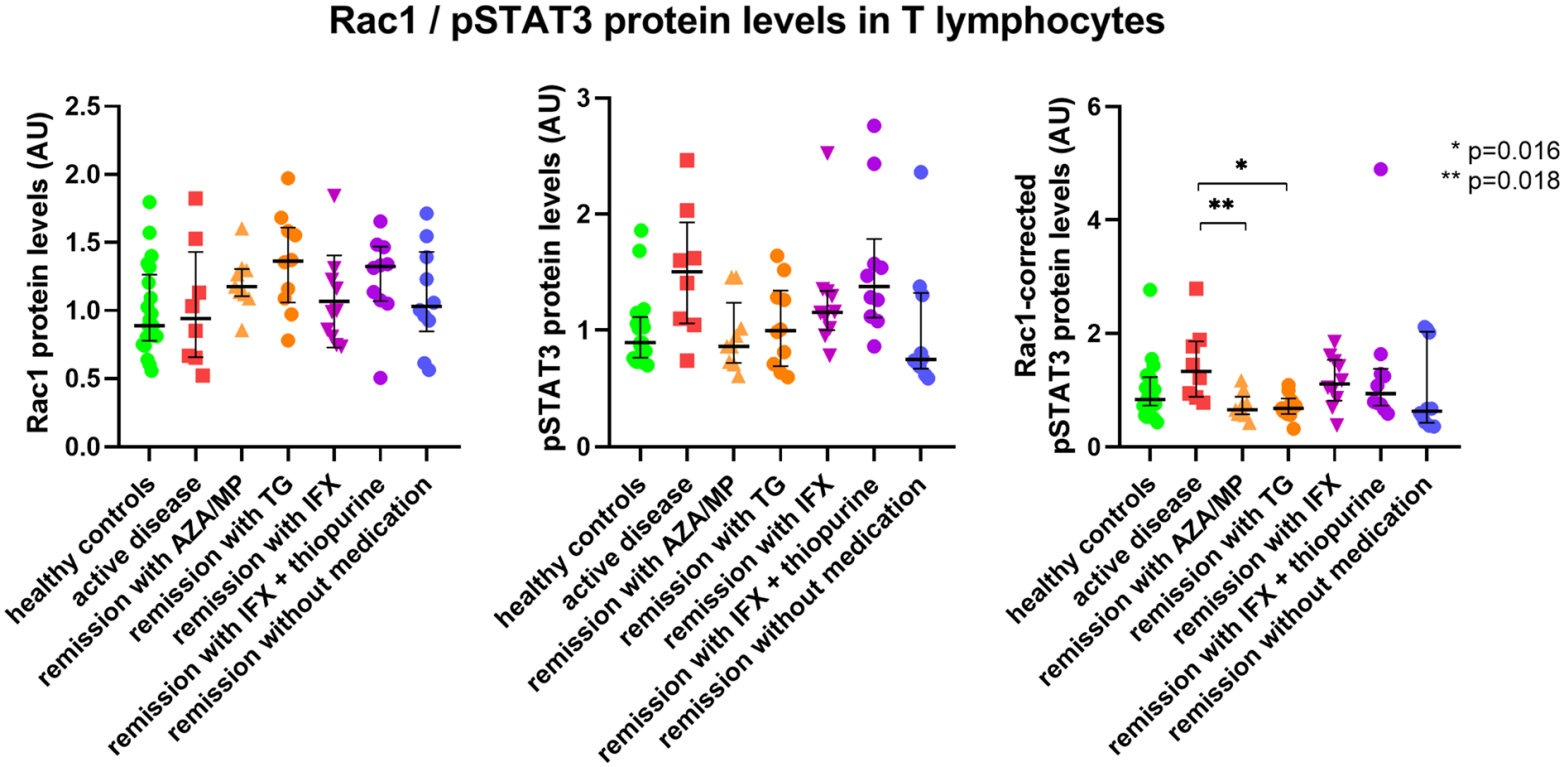
Rac1 (**A**) protein levels, pSTAT3 (**B**) protein levels and pSTAT3 corrected for Rac1 (**C**) in T lymphocytes of all drug-classified IBD groups. *AU* arbitrary units, *AZA* azathioprine, *IFX* infliximab, *MP* mercaptopurine, *TG* tioguanine.

Absolute Rac1 protein levels in the IBD patient groups in remission with thiopurine monotherapy on AZA/MP (median 1.18 AU) or TG (median 1.36 AU) did not differ from Rac1 protein levels in IBD patients with active disease (median 0.94 AU), (p > 0.01). Results are depicted in Fig. 4A. Absolute Rac1 protein levels in other drug-classified patients groups did not differ from the IBD groups in remission on AZA/MP or TG (p > 0.01), median 0.99 AU in the IFX monotherapy group, median 1.32 AU in the thiopurine/IFX combination group, and median 1.03 AU in the no therapy group, successively.

### pSTAT3 expression

A trend in reduction of absolute pSTAT3 protein levels in the IBD groups in remission with monotherapy AZA/MP (median 0.86 AU) or TG (median 0.99 AU) was observed when compared to IBD patients with active disease (median 1.50 AU, p = 0.024 and p = 0.054, respectively). Results are depicted in Fig. 4B. Absolute pSTAT3 protein levels of patient groups in remission with AZA/MP or TG monotherapy did not differ from other patient groups in remission with IFX (median AU 1.16), with thiopurine/IFX combination therapy (median 1.38 AU) or without IBD medication (AU 0.75) (p > 0.01).

### pSTAT3 corrected for Rac1

Rac1-corrected pSTAT3 protein levels in IBD patients groups on thiopurine monotherapy AZA/MP (median 0.66 AU) or TG (median 0.68 AU) were reduced compared to IBD patients with active disease with borderline significance (median 1.33 AU) (p = 0.018 and p = 0.016, respectively) (Fig. 4C). Compared to the other IBD patient groups in remission, no differences were found in Rac1-corrected pSTAT3 protein levels (p > 0.01). The median Rac1-corrected pSTAT3 protein levels of IBD patients in remission on AZA/MP or TG monotherapy were comparable to healthy subjects (median 0.84 AU).

## Discussion

In this explorative, observational study conducted in IBD patients subdivided into drug-classified groups, a remarkable, but theoretically predicted suppression of Rac1-corrected pSTAT3 protein levels in T lymphocytes of almost 50% were found in IBD patients in remission on thiopurine maintenance therapy, when compared to IBD patients with active disease.

This effect was observed both in the conventional thiopurine group (AZA/MP), as well as in the TG group. TG is known to have a different metabolic pathway compared to AZA/MP, with less enzymatic steps to eventually form the 6-TGN metabolites^27^. This finding strengthens the hypothesis that all thiopurines induce similar pharmacological immune suppressive effects, mediated by 6-TGTP induced Rac1-corrected pSTAT3 inhibition^28^.

Furthermore, 6-TGTP mediated Rac1 suppression in T lymphocytes has been previously described in vitro^20,21^. In this study, Rac1 suppression particularly concerned innate immunity, but adaptive T lymphocytes Rac1 suppression was observed as well^21^. The latter is believed to be the critical pharmacodynamic therapeutic effect of thiopurines in patients suffering from IBD^5,20^.

Additionally, it has previously been shown that the expression of Rac1 could potentially be used as a pharmacodynamic marker to optimize thiopurine therapy in IBD patients^18^. In that relatively small study (n = 11), however, the ‘on/off’ status of the Rac1 was taken into account by also measuring phosphorylated ezrin-radixin-moesin by Western blot analysis, a technically rather cumbersome method, not immediately suitable for daily practice in a hospital’s clinical laboratory setting.

With this approach, protein levels of both Rac1 and pSTAT3 could be measured in one flowcytometric analysis at the same time, thus simplifying its use in clinical practice. Even though STAT3 is not an immediate effector of Rac1, it has previously been reported that pSTAT3 may serve as a biomarker of the downstream effects of Rac1 inhibition^20,29^. To our knowledge, this is the first study in which Rac1 and pSTAT3 suppression in thiopurine therapy has been investigated in IBD patients. Additionally, the suggested potential pharmacodynamic marker was evaluated in different drug-classified groups of IBD patients and healthy subjects. It was observed that there were no differences between the other drug-classified groups and the healthy subjects, indicating that Rac1 and pSTAT3 suppression in T lymphocytes is thiopurine-specific.

A few remarks should be made regarding our study design. Due to relatively small patient groups and the use of the Bonferroni correction, no statistically significant differences (p < 0.01) were observed. However, this was a pilot study and no power analysis was performed to assess statistical significant differences. Another shortcoming of this explorative study was that our findings were based on a single measurement per patient in relatively small patient groups, not aiming to assess test accuracy in daily practice. A prospective longitudinal study with Rac1 and pSTAT3 measurements prior to and repeatedly during thiopurine treatment combined with evaluation of efficacy is warranted before Rac1 and pSTAT3 can be suggested as a pharmacodynamic marker in daily practice. Furthermore, measurement of Rac1 and pSTAT3 protein levels should ideally be performed in the (target) T lymphocytes present in the lamina propria of the affected intestinal epithelial. The T lymphocytes present in peripheral blood, as used in our approach, function as a surrogate marker. Lastly, patients in the group with active disease were not excluded due to prednisone or budesonide use as a means to achieve induction in anticipation of effective maintenance treatment. We cannot exclude the possibility that steroid use has a (concomitant) effect on the Rac1 and STAT3 pathway directly or via inhibition of cytokines.

Interestingly, combined thiopurine and anti-TNF therapy did not result in a statistically significant decrease of Rac1-corrected pSTAT3 protein levels when compared to the active disease patients. Thiopurine doses (mg/kg bodyweight) were comparable between the thiopurine monotherapy groups and the combination therapy group, which therefore could not explain the lack of decrease in Rac1-corrected pSTAT3 protein levels. We have no suitable explanation for this observation and further research is needed to elucidate this. Anti-TNF biologicals are widely prescribed immune suppressive agents. Synergistic effects on dual immune suppression have been described for combination therapy of IFX with thiopurines^8^. This combination therapy may help to minimize immunogenicity and improve clinical outcomes, because of a suspected positive pharmacokinetic effect of thiopurines on anti-TNF treatment. In line with these findings, optimal dosing of thiopurines in combination with anti-TNF may be different than currently used for monotherapy with thiopurines.

Notably, in the active disease patient group, only patients with mild and moderate disease activity were included. However, this is consistent with the indication to start thiopurine therapy. It is therefore likely to assume that our small patient group is representative for thiopurine patients in general.

Regarding the late onset of action of thiopurines and the relatively high percentage of adverse drug events potentially leading to early therapy failure, an early pharmacodynamic marker, such as Rac1-corrected pSTAT3 protein levels, might probably contribute to improving therapeutic benefit for IBD patients by personalizing thiopurine therapy.

In conclusion, in this explorative study we demonstrated that Rac1-corrected pSTAT3 protein levels in T-lymphocytes seem a specific pharmacodynamic marker for thiopurine therapy in IBD patients, and may be a potential tool to optimize thiopurine therapy in clinical practice.

## Supplementary Information

Supplementary Information.

## Data Availability

Raw data were generated at Zuyderland Medical Centre. Derived data supporting the findings of this study are available from the corresponding author DD on request.
