# Supplementary material for: A report on the potential of Rac1/pSTAT3 protein levels in T lymphocytes to assess the pharmacodynamic effect of thiopurine therapy in Inflammatory Bowel Disease patients
Source: Sci Rep. 2022 Sep 22;12:15806. doi: 10.1038/s41598-022-20197-5 (PMC9500076; doi:10.1038/s41598-022-20197-5)

**SUPPLEMENTAL INFORMATION**


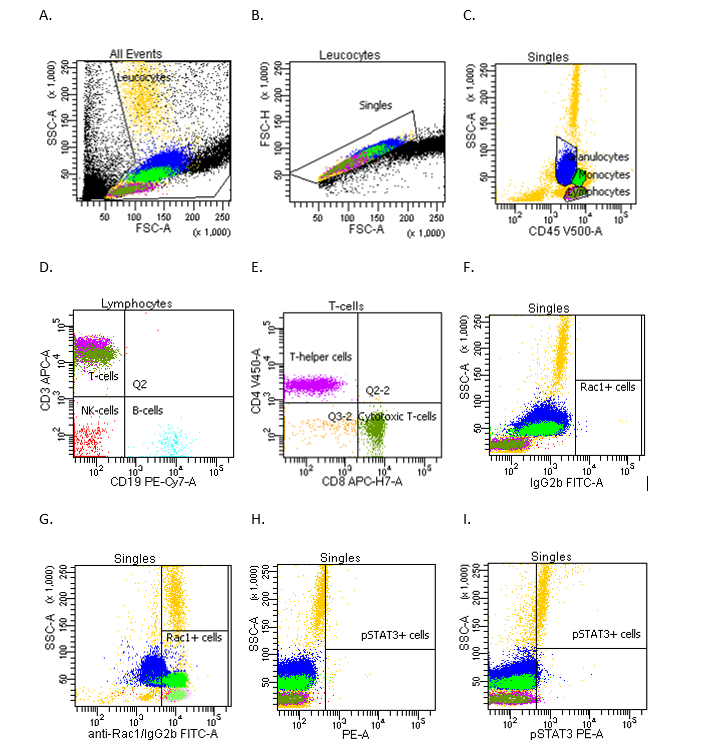


**Supplemental figure 1**: The gating procedure for differentiation of leukocyte subpopulations.

A: Leukocyte isolation, B: Isolation of single cells, C: Gating procedure of granulocytes, monocytes and lymphocytes, D: Subdivision of lymphocytes into T cells, B cells and ‘Not B or T cells’, E: subdivision of T lymphocytes into T helper cells and cytotoxic T cells.
F and H: Negative controls for presence of Rac1 and pSTAT3 were tested, respectively. G and I: presence of Rac1 and pSTAT3 are shown, respectively.

**Supplemental 2:** examples of MFI calculation of Rac1 and pSTAT3 from the corresponding histograms, including the calculation to Arbitrary Units.

**A. Raw flow cytometric data of the Rac1 tube of example patient 2. The Mean Fluorescent Intensity (MFI) of T cells is shown in red.**

**
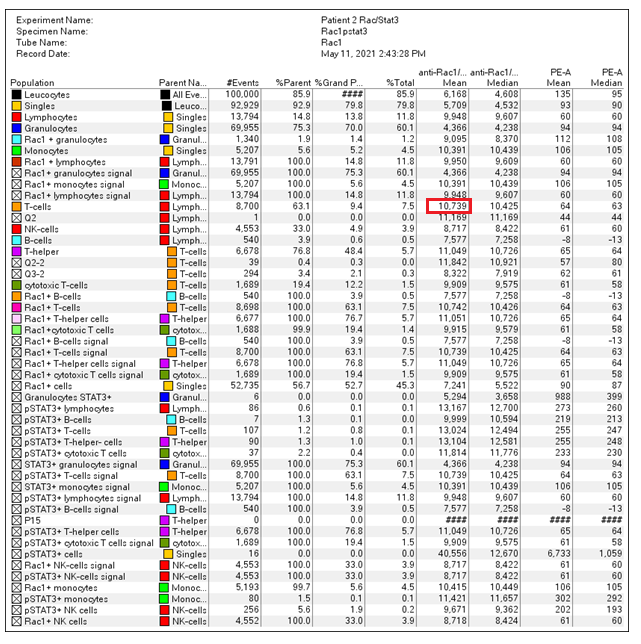
**

**B. Raw flow cytometric data of the pSTAT3 tube of example patient 2. The Mean Fluorescent Intensity (MFI) of T cells is shown in red.**

**
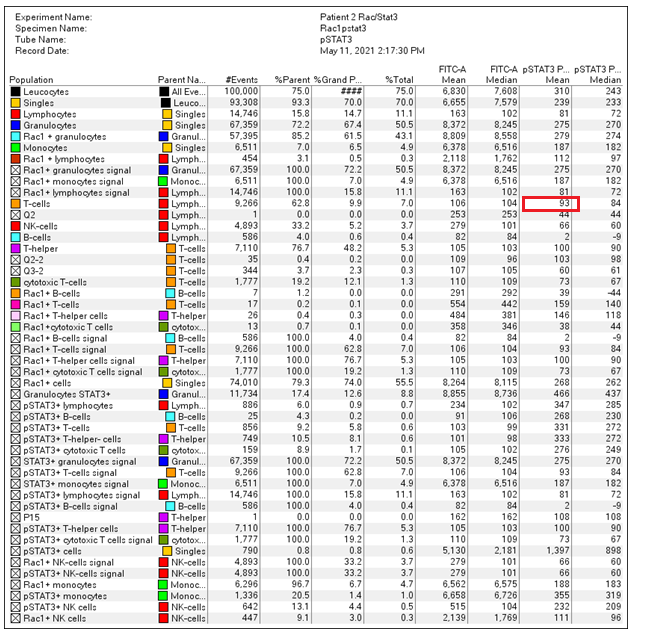
**

**C. Calculation from raw data into arbitrary units (AU)**

|  | **Rac_**  **MFI** | **Normalized _rac (AU)** | **pSTAT3_**  **MFI** | **normalized_**  **pSTAT3 (AU)** | **pSTAT3/Rac_**  **MFI** | **normalized_**  **ratio (AU)** |
| --- | --- | --- | --- | --- | --- | --- |
| Mean of healthy subjects* | 9086 | 1 | 97 | 1 | 0,014 | 1 |
| Example Patient 2 | 10739^a^ | 1,18 | 93^b^ | 0,96 | 0,0087 | 0,62 |

*mean data of all healthy subjects, therefore not to be shown as raw data files.

^a^raw data file shown in Figure 2A

^b^raw data file shown in Figure 2B

**D. Example of traceability of raw data into graphs**


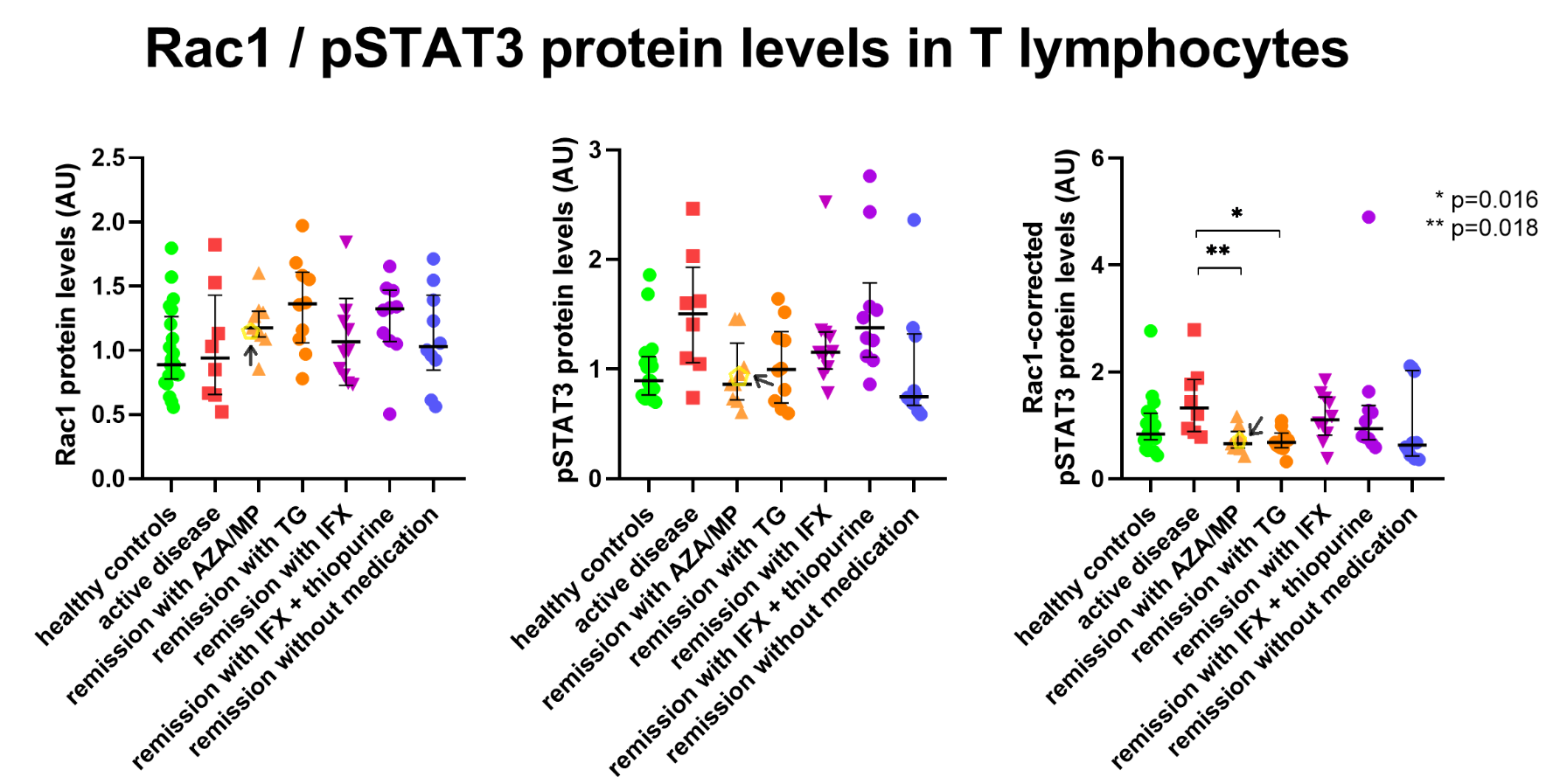


**Supplemental raw data**
All raw data are shown below in the following order:

1) negative tube
2) Rac1 tube
3) pSTAT3 tube

**EXAMPLE PATIENT 1**

1) negative tube


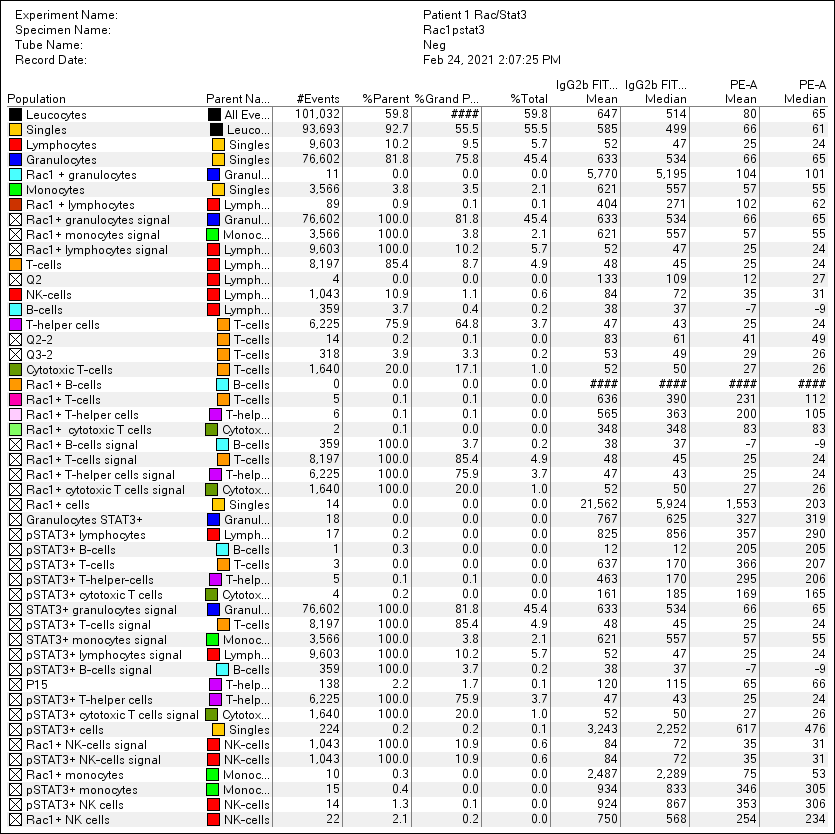


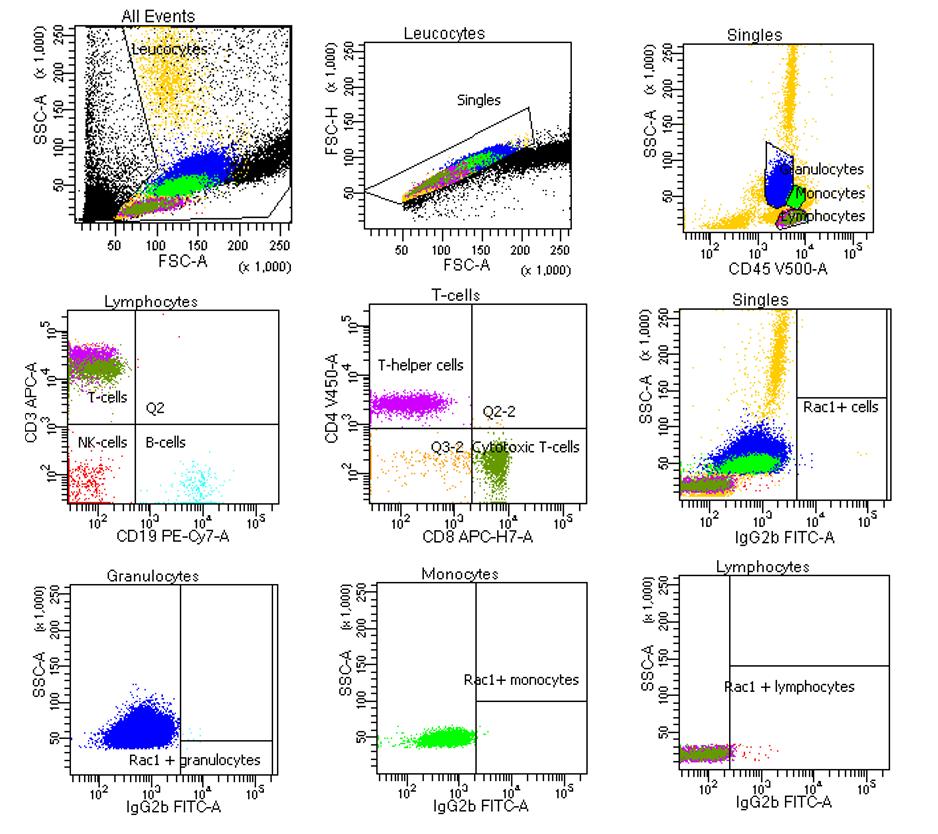


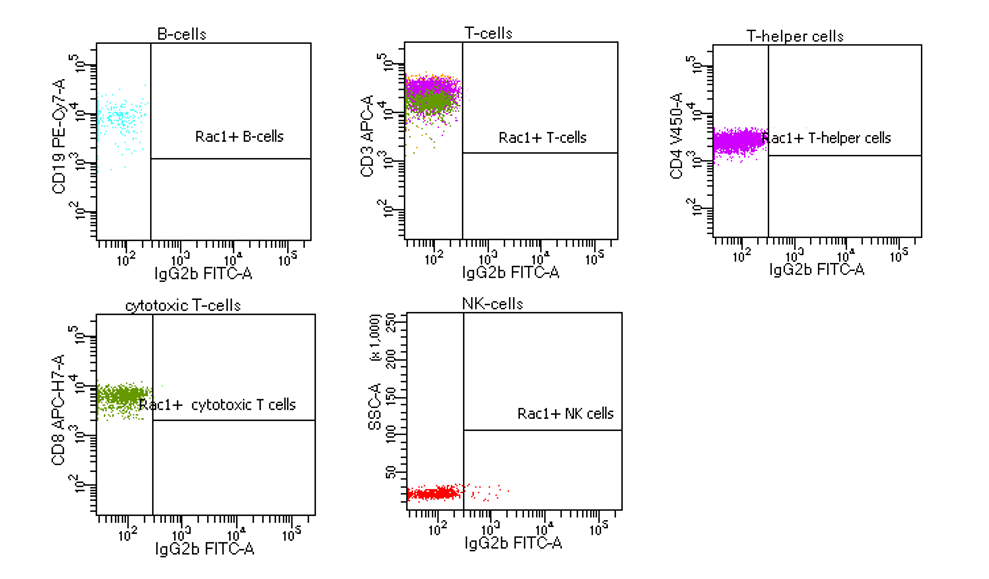


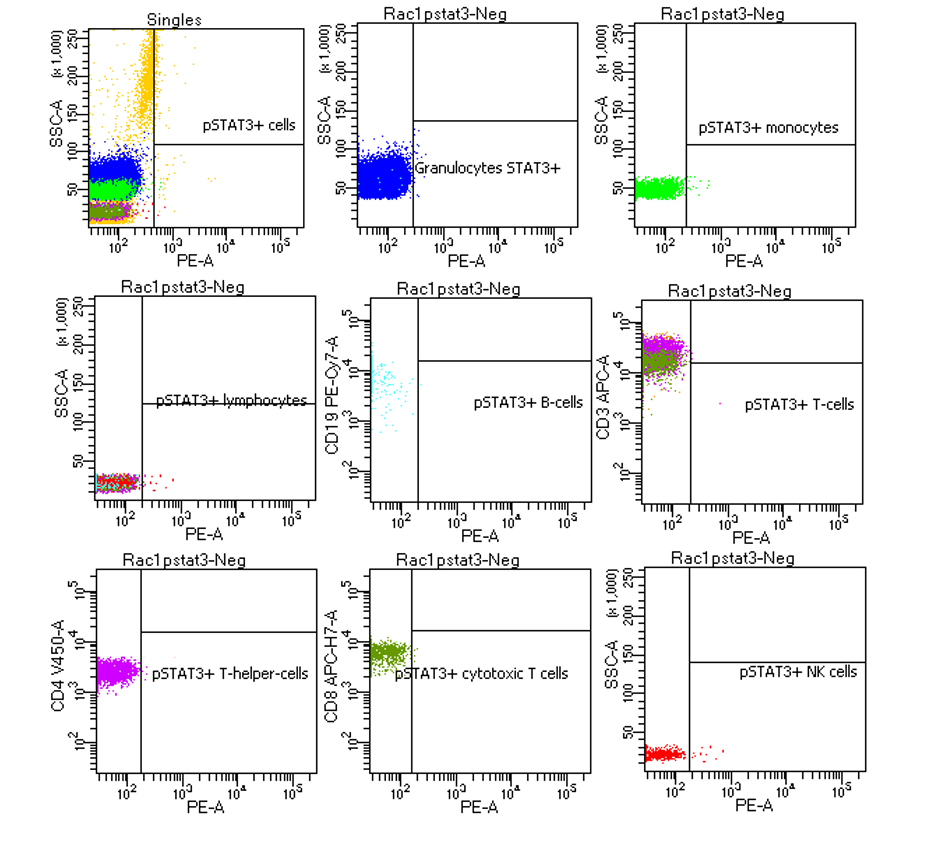


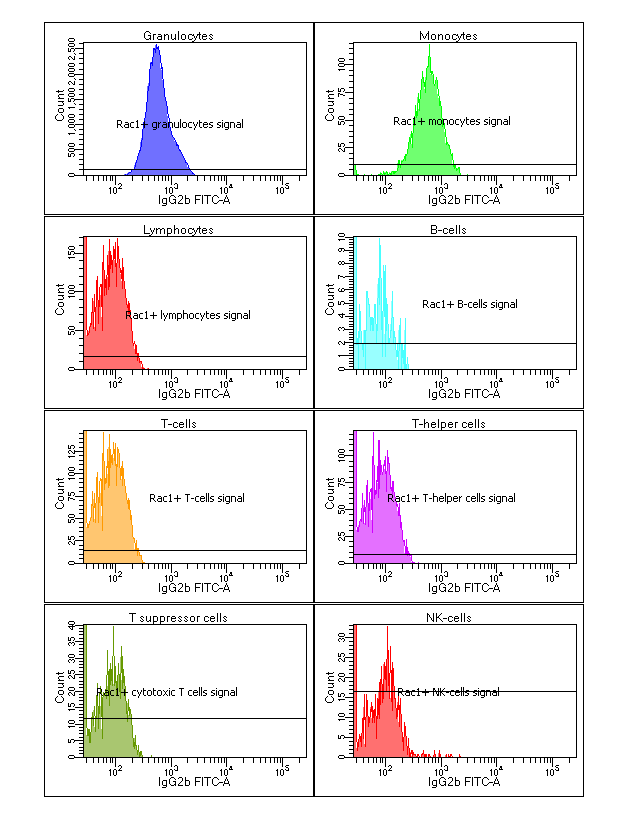


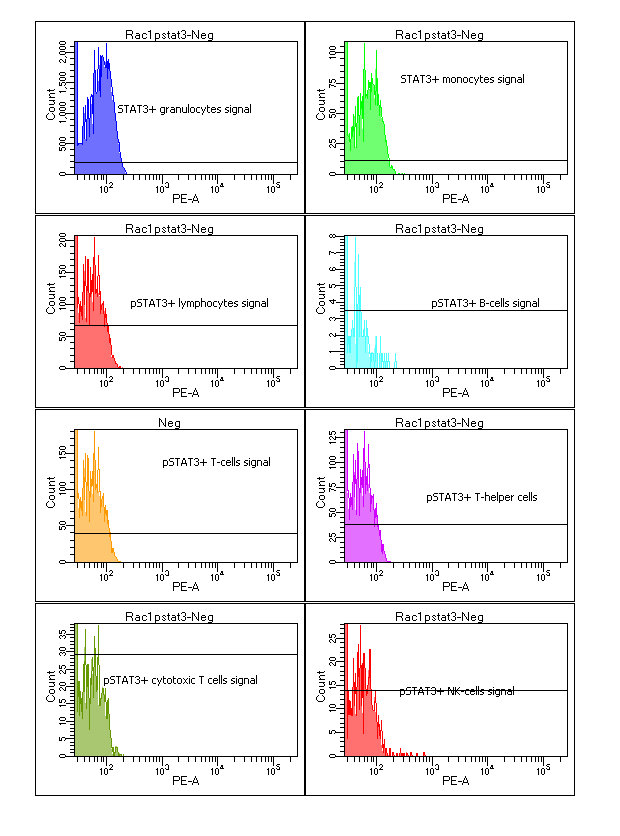


2) Rac1 tube


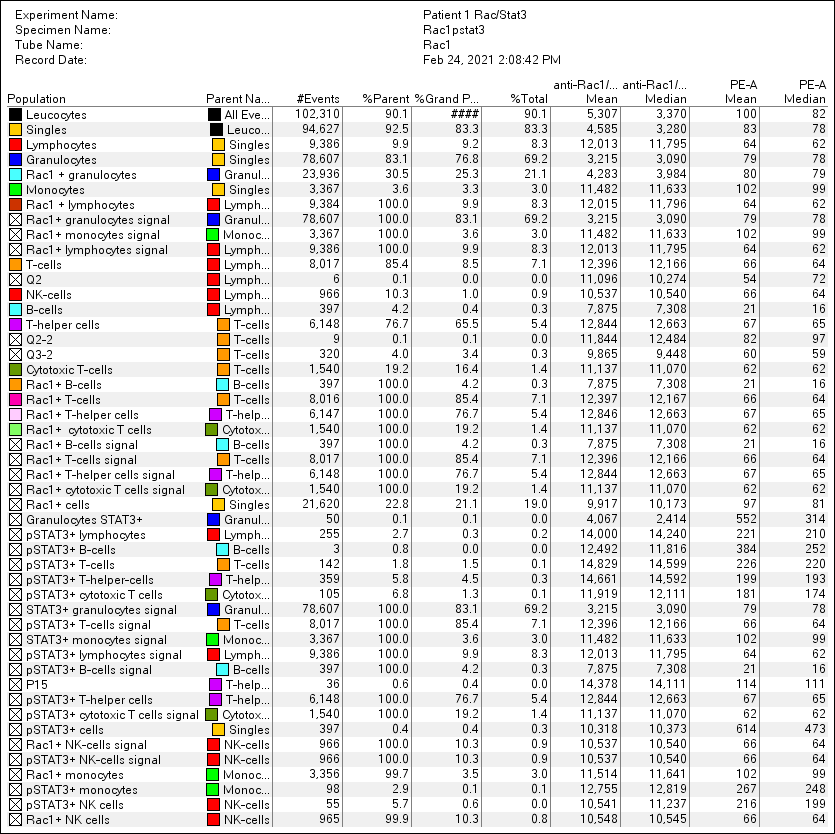


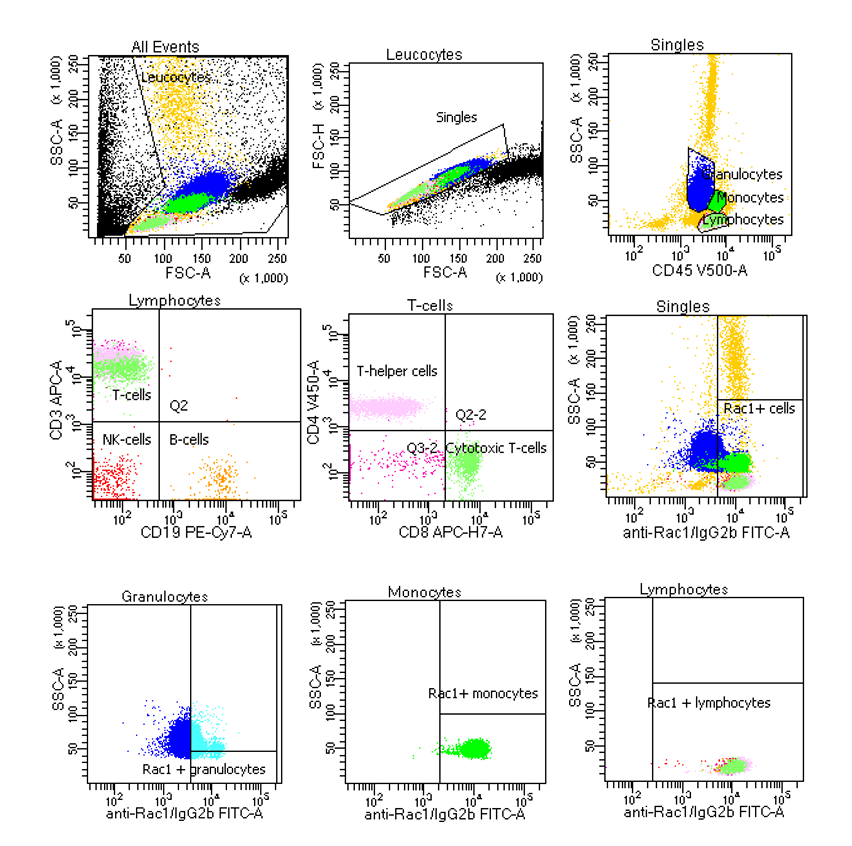


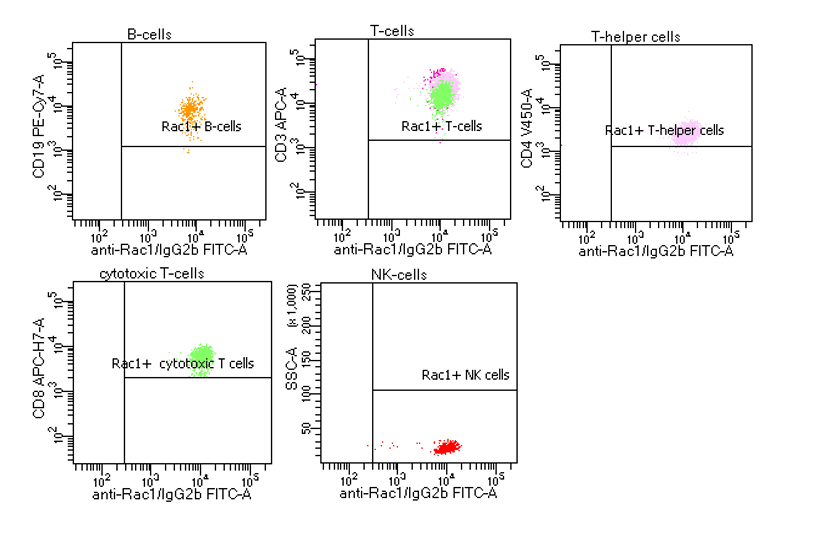


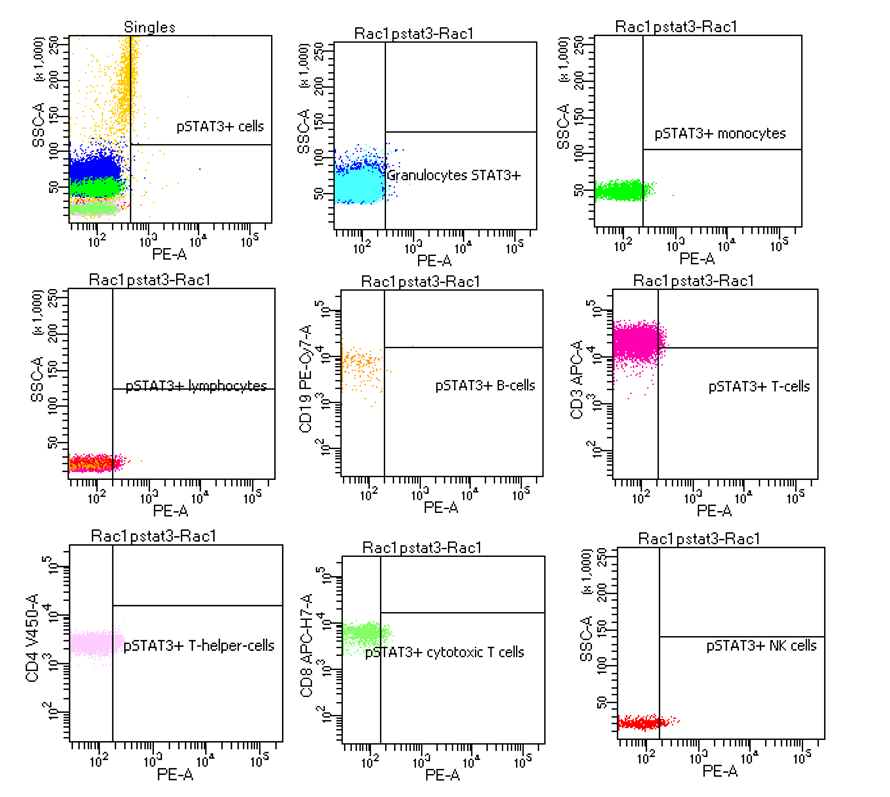


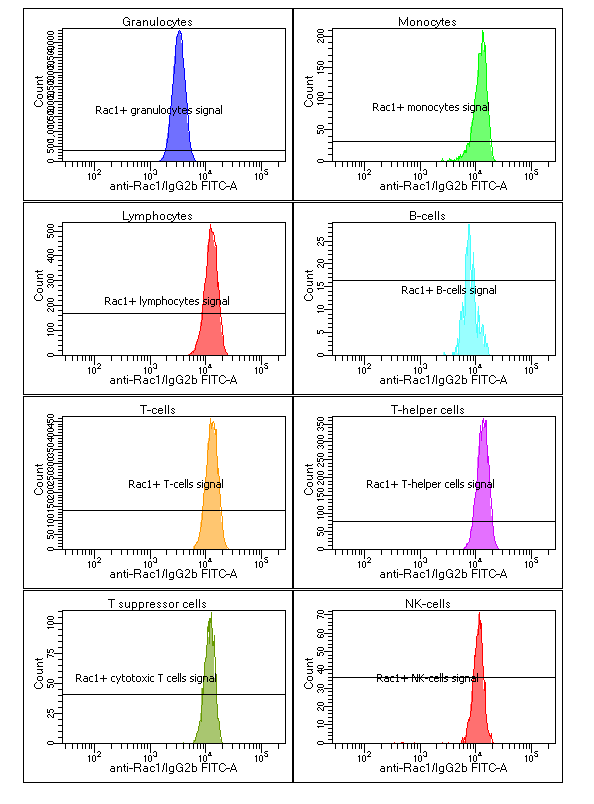


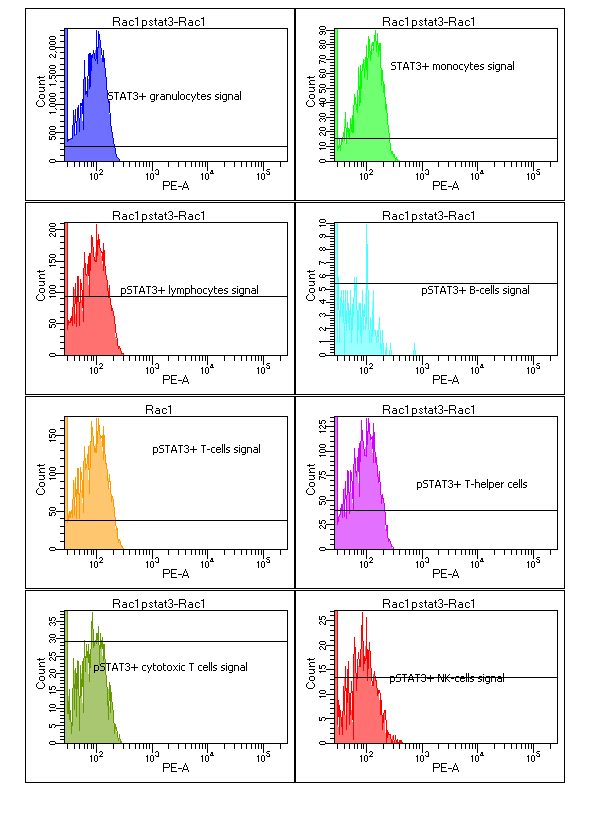


3) pSTAT3 tube


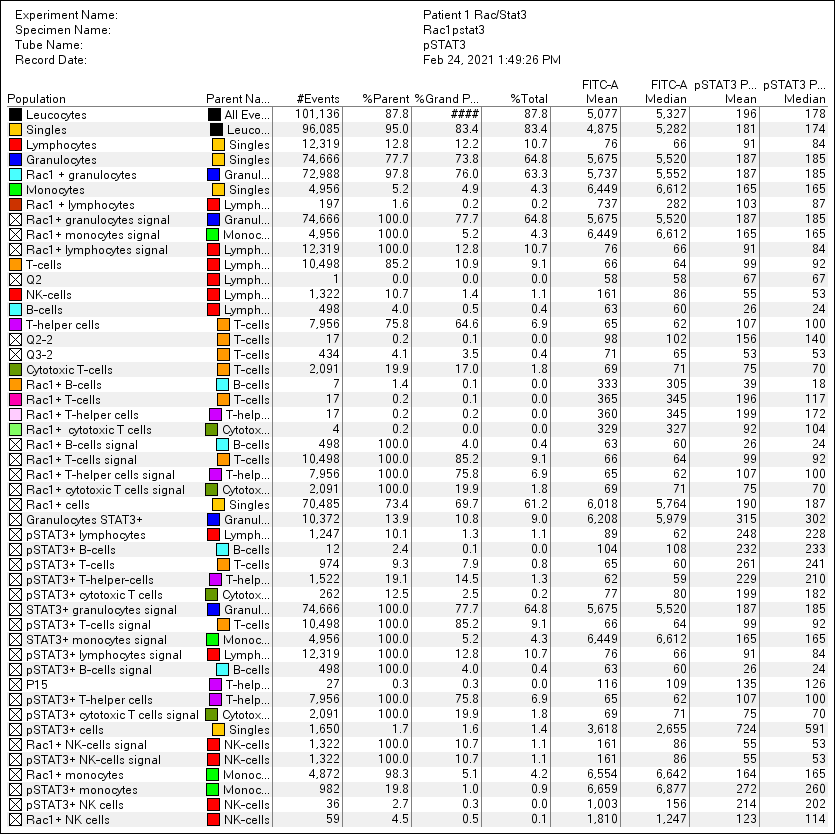


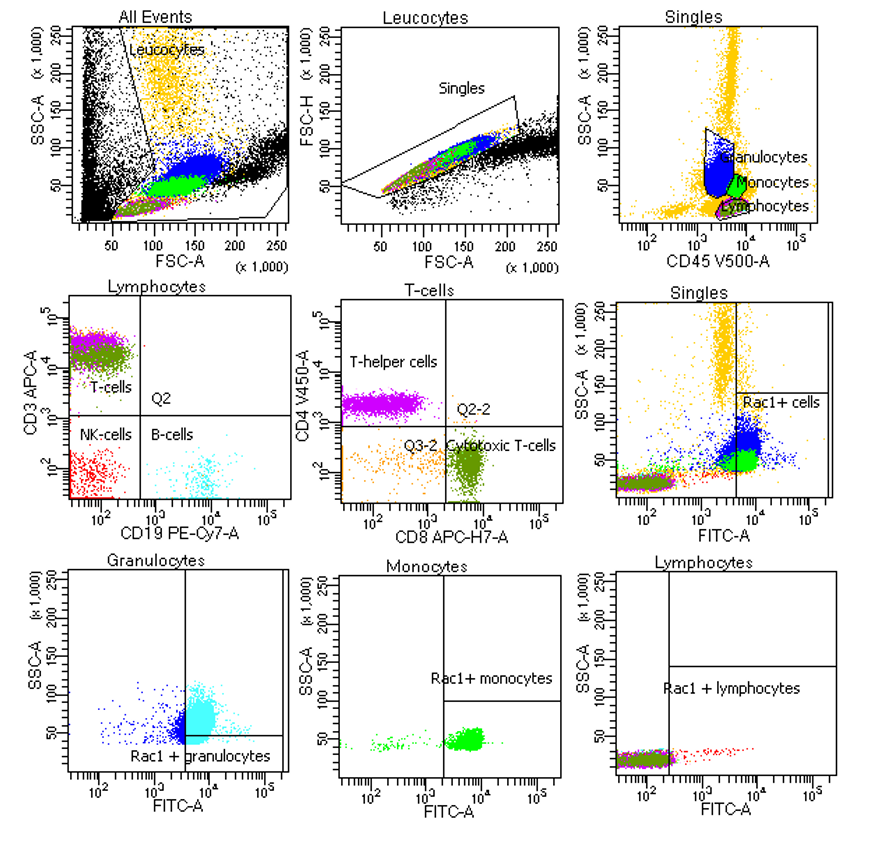


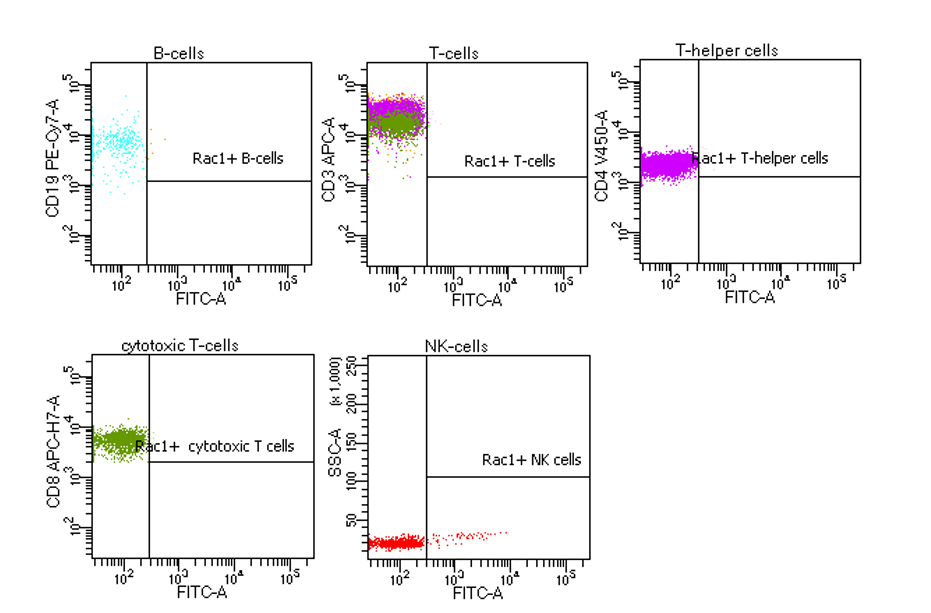


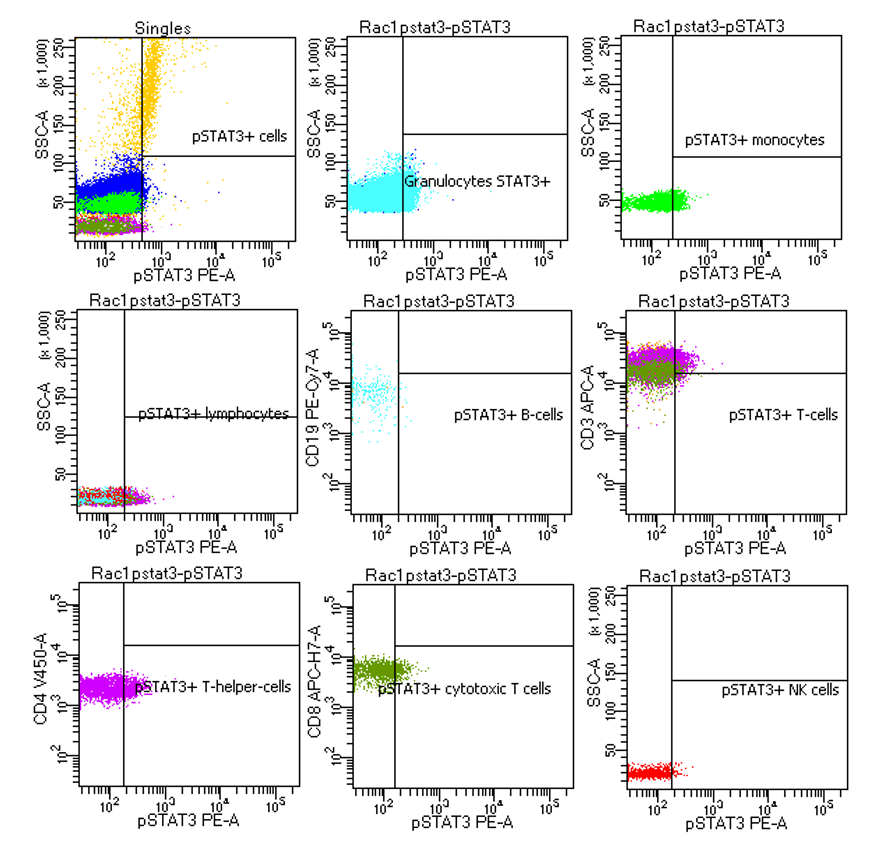


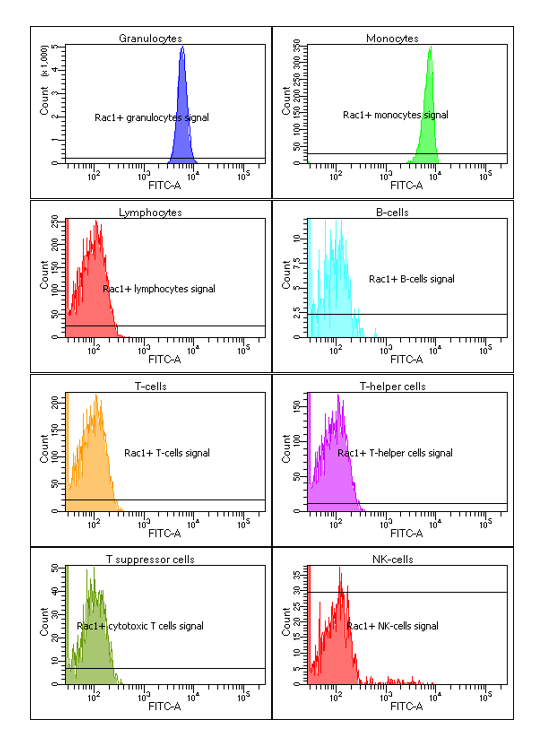


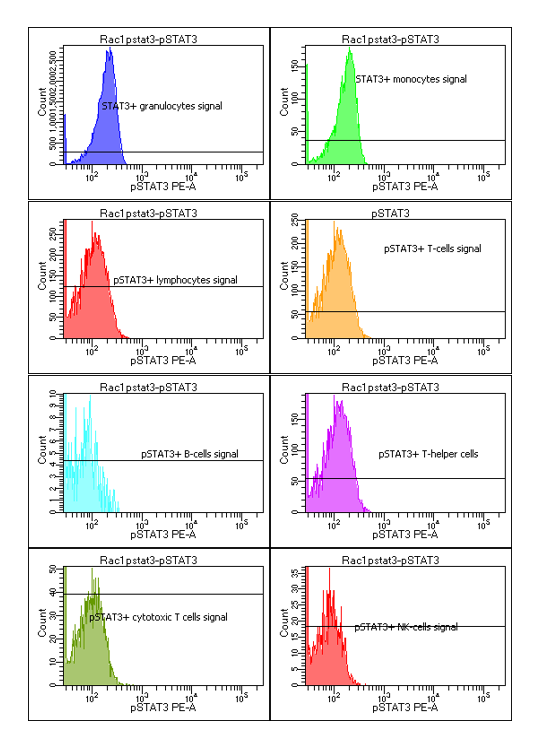

Supplement: Supplementary file 1 — Supplementary Information. [file 41598_2022_20197_MOESM1_ESM.docx]
